# Immunogenicity Risk Assessment of Spontaneously Occurring Therapeutic Monoclonal Antibody Aggregates

**DOI:** 10.3389/fimmu.2022.915412

**Published:** 2022-07-27

**Authors:** Michael D. Swanson, Shantel Rios, Sarita Mittal, George Soder, Vibha Jawa

**Affiliations:** ^1^ Pharmacokinetics, Pharmacodynamics and Drug Metabolism, Merck Research Laboratories, Merck & Co., Inc., Kenilworth, NJ, United States; ^2^ Analytical R&D, Merck Research Laboratories, Merck & Co., Inc., Kenilworth, NJ, United States; ^3^ Nonclinical Disposition and Bioanalysis, Bristol Myers Squibb, Princeton, NJ, United States

**Keywords:** aggregation, monoclonal, immunogenicity, THP-1 activation assay, innate & adaptive immune response, immunogenicity risk assessment, human PBMC

## Abstract

Aggregates of therapeutic proteins have been associated with increased immunogenicity in pre-clinical models as well as in human patients. Recent studies to understand aggregates and their immunogenicity risks use artificial stress methods to induce high levels of aggregation. These methods may be less biologically relevant in terms of their quantity than those that occur spontaneously during processing and storage. Here we describe the immunogenicity risk due to spontaneously occurring therapeutic antibody aggregates using peripheral blood mononuclear cells (PBMC) and a cell line with a reporter gene for immune activation: THP-1 BLUE NFκB. The spontaneously occurring therapeutic protein aggregates were obtained from process intermediates and final formulated drug substance from stability retains. Spontaneously occurring aggregates elicited innate immune responses for several donors in a PBMC assay with cytokine and chemokine production as a readout for immune activation. Meanwhile, no significant adaptive phase responses to spontaneously occurring aggregate samples were detected. While the THP-1 BLUE NFκB cell line and PBMC assays both responded to high stress induced aggregates, only the PBMC from a limited subset of donors responded to processing-induced aggregates. In this case study, levels of antibody aggregation occurring at process relevant levels are lower than those induced by stirring and may pose lower risk *in vivo*. Our methodologies can further inform additional immunogenicity risk assessments using a pre-clinical *in vitro* risk assessment approach utilizing human derived immune cells.

## Introduction

Therapeutic antibodies are prone to form aggregates during manufacturing, handling, and storage. A combination of different patient and drug characteristics factor into the immunogenicity of protein-based therapeutics, but aggregates have been implicated in provoking immune responses (1). These responses include the formation of anti-drug antibodies which have the potential to impact drug efficacy as well as patient safety. For example, tungsten-induced aggregates of recombinant human erythropoietin were implicated in cases of pure red cell aplasia in patients (2).

Although stress induced aggregates of monoclonal antibodies have shown increased immunogenicity *in vitro*, the risks of more relevant, lower levels of aggregates found in unstressed antibody products are unknown. For example, steps during the purification process may promote the formation of antibody aggregates which may end up in the final product (3, 4). In addition, aggregation can occur during and after drug fill-finish. Shear stress, agitation, mixing speed and filtration can impact aggregate formation during fill-finish (4, 5). Post fill examples of antibody aggregate formation include product temperature excursions due to improper storage and mishandling such as dropping of vials (6).

While peripheral blood mononuclear cells (PBMC) have been the primary *in vitro* cell based model for assessing *in vitro* immunogenicity risk, the utility of cell-line based models remains unknown. The monocyte-like cell lines Mono-Mac-6 and THP-1 have been shown to respond to intravenous immunoglobulin aggregates *in vitro* as evident by an increased production of TNF-α, IL-1β, IL-6, IL-8, and IL-10 (7). A commercially available derivative of THP-1 called THP-1 BLUE NFκB has a simple colorimetric readout for immune activation and potential for a higher throughput format that may be advantageous to evaluate immunogenicity posed by antibody aggregates.

While most studies on aggregate immunogenicity are performed with stressed antibodies, here we investigate the immunogenicity of spontaneously occurring monoclonal antibody aggregates using PBMC and the cell line THP-1 BLUE NFκB.

## Materials and Methods

### Antibodies

mAb1 is a human, hinge-stabilized IgG4. Stability testing at accelerated and stressed temperatures revealed that mAb1 has a propensity to aggregate. Remicade (infliximab) and Herceptin (trastuzumab) were reconstituted with water and stored at -80°C. Stressed positive controls were obtained by diluting the material to 1 mg/mL with saline and stirring with a PTFE magnetic stir bar for 1 to 3 days at 700 rpm stirring speed and at room temperature. All antibodies used in the assay were confirmed for low endotoxin levels.

### Aggregate Characterization

Sub-visible particles were characterized using an MFI 5100 (Protein Simple) instrument equipped with a 400 µm flow cell. The particle size range that can be characterized is from 2 to 300 microns. A 5 µm size standard was run prior to testing. Samples were diluted from 0.01 to 1 mg/mL in water. A sample volume of 900 µL was analyzed per sample.

For measuring aggregates *via* light obscuration, a HIAC 9703+ instrument equipped with a 10 mL syringe was used to analyze mAb1 samples, according to the manufacturer’s instructions. Briefly, samples were pooled into a 50mL polypropylene sterile container and allowed to de-gas for 30 minutes. Environment test with water was conducted prior to sample testing to establish a particle free system. Five runs of one mL each were performed per sample. The first two runs were discarded, and the final three runs were averaged to yield the result.

### Primary Cells and Cell Line Experiments

**Figure 1:**
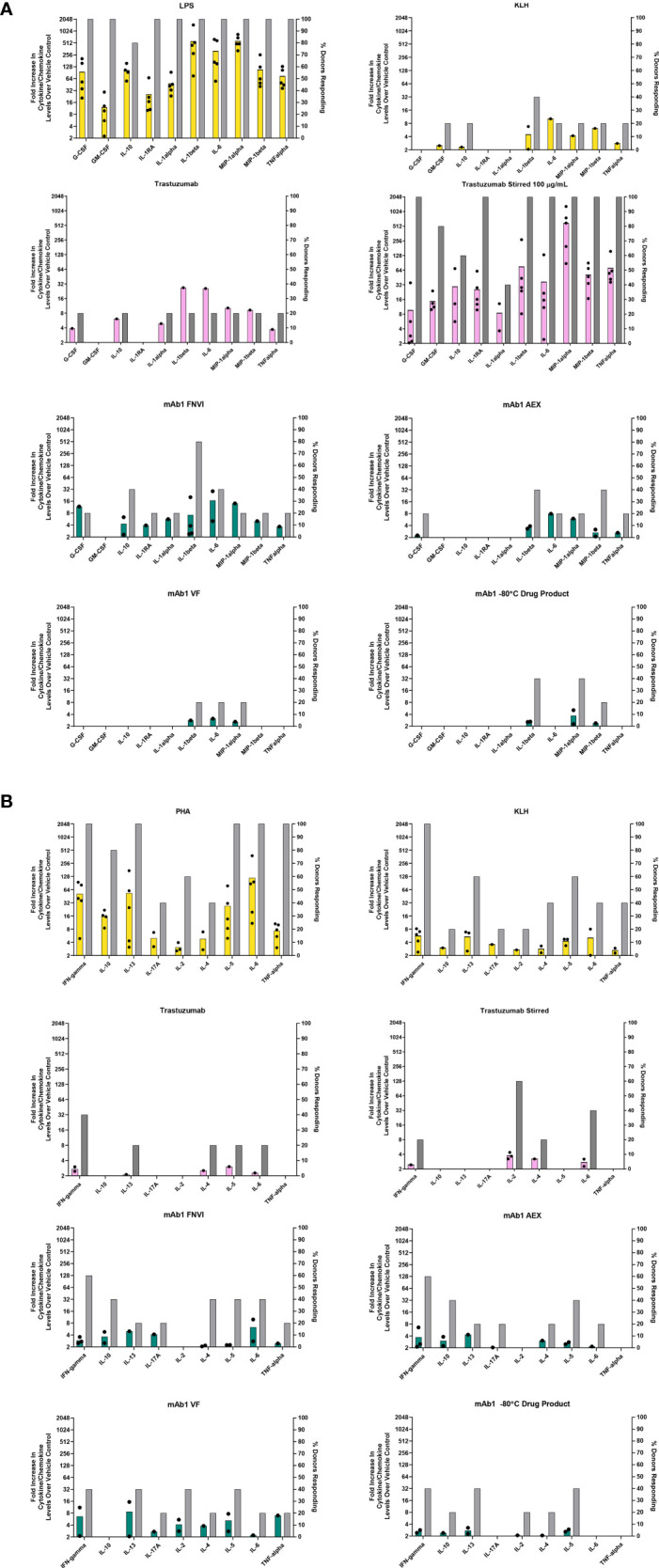
*In vitro* PBMC responses to spontaneously occurring antibody aggregates obtained from process intermediates. Colored bar graphs correspond to the left Y-axis and represent the mean of positive donor responses (fold increase ≥ 2 is considered positive). Black dots show the individual donors that had a positive response. The grey bars correspond to the right Y-axis and represent the proportion of donors with positive responses to a particular analyte. **(A)** Innate immune responses measured at 20 hrs. **(B)** Adaptive immune responses measured on day 7. Data are from 5 donors.

**Figure 2:**
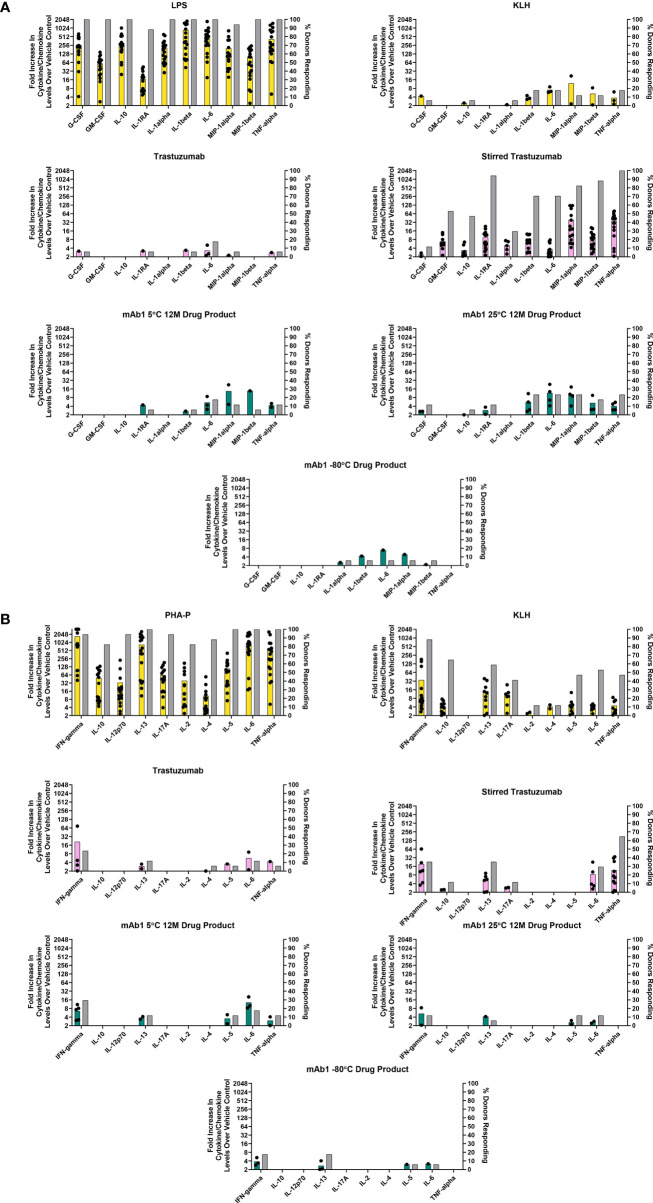
*In vitro* PBMC responses to spontaneously occurring antibody aggregates obtained from stability samples stored at different temperatures. Colored bar graphs correspond to the left Y-axis and represent the mean of positive donor responses (fold increase ≥ 2 is considered positive). Black dots show the individual donors that had a positive response. The grey bars correspond to the right Y-axis and represent the proportion of donors with positive responses to a particular analyte. **(A)** Innate immune responses measured at 20 hrs. **(B)** Adaptive immune responses measured on day 7. Data are from 17 donors.

Cryopreserved PBMC were obtained from healthy donors and were procured *via* Precision for Medicine. Cells were thawed and resuspended at a concentration of 4.4 x 10^6 cells/mL in RPMI-1640 media (ThermoFisher Catalog# 61870-036) containing 10% heat inactivated FBS (ThermoFisher Catalog# 10082-147), 10 mM HEPES (ThermoFisher Catalog#15630-080), 3.7 mM GlutaMAX(ThermoFisher Catalog# 35050-061), 1 mM sodium pyruvate (ThermoFisher Catalog# 11360-070), 55 µM beta-mercapto-ethanol (ThermoFisher Catalog# 21985-023), 100 µg/mL normocin (*Invivo*Gen Catalog# ant-nr-1), 100 units/mL penicillin and 100 µg/mL streptomycin (ThermoFisher Catalog# 15140-122) for the results in **Figure 1**. X-Vivo 15 media (Lonza Catalog# 04-418Q) was used in **Figure 2**. One hundred eighty µL of the cell suspension (7.92 x 10^5 cells per well) was added to the wells of a flat bottom, tissue culture treated, 96-well plate. Test material was added at a volume of 20 µL and incubated at 37°C with 5% CO_2_. The final concentration of antibodies and Keyhole limpet hemocyanin (KLH) were 100 µg/mL with a final volume of 200 µL per well. Samples were taken at 20 hrs post addition for an innate response measurement and at day 7 for adaptive response measurement. Samples were stored at -80°C until analysis.

The cell line THP-1 BLUE NFκB was purchased from *Invivo*Gen (Catalog# thp-nfkb). Cells were cultured in 88.8% RPMI-1640 media with GlutaMAX and HEPES (ThermoFisher Catalog# 72400-047), 10% heat inactivated FBS with 100 µg/mL normocin (*Invivo*Gen Catalog# ant-nr-1), 100 units/mL penicillin and 100 µg/mL streptomycin (ThermoFisher Catalog# 15140-122). A volume of 180 µL of cells at a concentration of 1.1 x 10^6 cells/mL were added to the wells of a flat bottom, tissue culture treated, 96-well plate. Test material was added at a volume of 20 µL and incubated at 37°C with 5% CO_2_. FSL-1 (*In vivo*Gen Catalog# tlrl-fsl) and LPS (Sigma Catalogt# LPS25) were used as positive controls. After 20 hrs of incubation, the cells were centrifuged at 300 x g for 5 minutes and supernatant samples were removed.

### Cytokine/Chemokine/Reporter Gene Response Measurement

SEAP activity was measured by adding 20 µL of cell culture supernatant to 180 µL of QUANTI-Blue (*In vivo*Gen Catalog# rep-qbs) in a 96 well plate. The plate was incubated for 2 hrs at 37°C before measuring the absorbance at 655 nm. Customized luminex kits were purchased from Millipore Sigma (Catalog# HYCTOMAG-60K) and adapted to a 384 well plate format based on previously described methods (8).

## Results

### Analytical Characterization of Antibody Aggregates

Upstream processing material from three different stages of the purification process for the monoclonal antibody mAb1 (described further in the Materials and Methods section) were utilized in this study: material from a 0.2 µm filtered, neutralization/viral inactivation step (FNVI) that occurred after a protein A chromatography step, mAb1 material following an anion exchange chromatography step (AEX), and mAb1 material following a virus filtration step (VF). Microflow imaging was performed to assess the amount of antibody aggregates present. The highest levels of aggregates were observed from the FNVI samples while AEX and VF samples had similar levels (**Table 1**). Particles were observed despite a 0.2 µm filtration that was performed on the material from each step. This could be due to submicron aggregates that were present after the filtration step which acted as seed material for the formation of larger aggregates. Our rationale for using the upstream process material was that these spontaneous forming aggregates would be better representative in terms of size and size distribution than material from highly stressed conditions.

**Table 1:** Microflow imaging analysis of antibody aggregates: Comparison of spontaneously forming aggregates from upstream processing.

Sample	2-5 µm	5-10 µm	10-25 µm	25-50 µm
mAb1 FNVI	11,121	3,729	1,380	60
mAb1 AEX	1,526	472	224	25
mAb1 VFP	1,824	427	158	16
Trastuzumab	1,010	330	178	6
Trastuzumab Stirred	502,730	83,950	11,886	36

Sub-visible particles were characterized using an MFI 5100 (Protein Simple) instrument equipped with a 400 µm flow cell. The particle size range that can be characterized is from 2 to 300 microns. A 5 µm size standard was run prior to testing. Samples were diluted from 0.01 to 1 mg/mL in water. A sample volume of 900 µL was analyzed per sample. Per 1 mg.

For a relative comparison, trastuzumab was reconstituted and was stored at -80°C or alternatively was diluted to 1 mg/mL and stir stressed to induce high levels of antibody aggregation. The aggregate content in unstressed trastuzumab was as high as the process related mAb1 samples. This high level can be due to inadequate storage conditions (freezing the material is not recommended by the manufacturer). Trastuzumab was also stir stressed to induce aggregates and contained approximately 37-fold more particles (≥2µm size) than the mAb1 FNVI material.

Final formulated drug substance forms of mAb1 were also tested for the formation of aggregates following storage at elevated temperatures. The sizes and amounts of spontaneously formed aggregates were determined by microflow imaging and light obscuration *via* HIAC. The mAb1 drug product stored at 5°C and 25°C was associated with approximately 2- and 2.9-fold more particles (≥2µm size) than the mAb1 drug product stored at -80°C (**Table 2**). HIAC found fewer aggregates in the mAb1 drug product stored at -80°C than what was observed with microflow imaging. Increased levels of aggregates were observed in the mAb1 drug product stored at elevated temperatures (**Table 3**).

**Table 2:** Microflow imaging analysis of antibody aggregates: Comparison of spontaneously forming aggregates from drug stability testing.

Sample	2-5 µm	5-10 µm	10-25 µm	25-50 µm
Trastuzumab	1,350	114	33	0
Trastuzumab Stirred	2,080,945	83,140	13,342	1,790
Infliximab	832	16	33	0
Infliximab Stirred	18,183,134	3,317,473	455,074	6,346
mAb1 -80°C	399	39	6	0
mAb1 5°C 12M	846	114	16	0
mAb1 25°C 12M	1,181	86	16	1

Sub-visible particles were characterized using an MFI 5100 (Protein Simple) instrument equipped with a 400 µm flow cell. The particle size range that can be characterized is from 2 to 300 microns. A 5 µm size standard was run prior to testing. Samples were diluted from 0.01 to 1 mg/mL in water. A sample volume of 900 µL was analyzed per sample. Per 1 mg.

**Table 3:** Light Obscuration Results.

Sample	≥10 µm	≥25 µm
mAb1 -80°C	7	0
mAb1 5°C 12M	>1.80E4	>1.8E4
mAb1 25°C 12M	>1.80E4	>1.80E4

For measuring aggregates via light obscuration, a HIAC 9703+ instrument equipped with a 10 mL syringe was used to analyze mAb1 samples, according to the manufacturer’s instructions. Briefly, samples were pooled into a 50mL polypropylene sterile container and allowed to de-gas for 30 minutes. Environment test with water was conducted prior to sample testing to establish a particle free system. Five runs of one mL each were performed per sample. The first two runs were discarded, and the final three runs were averaged to yield the result. Per 100 mg.

Additional preparations of infliximab and trastuzumab that were stir stressed or stored at -80°C were also analyzed. Again, the storage after reconstitution likely resulted in generating particles that were detected in the unstressed trastuzumab and infliximab (**Table 2**).

### Innate and Adaptive PBMC Responses to Aggregate Containing Process Intermediate Samples

The human PBMC were challenged with mAb1 sample intermediates obtained at different stages of process development (**Table 1**). We decided to evaluate these materials to see if spontaneously occurring aggregates are immunogenic in *in vitro* assays. PBMC were incubated with mAb1 samples from the three different process intermediates. For relative comparison, trastuzumab, stirred trastuzumab, KLH, and LPS were included (**Figure 1A**).

For innate phase response evaluations, cytokines and chemokines levels were determined from samples obtained 20 hrs post addition of sample and the number of donors with positive responses were determined as well. We considered a positive response being a measurement of two-fold and greater over the vehicle treated control cells. As expected, all five donors had high cytokine/chemokine responses indicative of an innate phase response 20 hrs post addition of the innate TLR4 ligand LPS (**Figure 1A**). The protein KLH contains numerous neoantigen derived T cell epitopes and can be used as a positive control for T cell mediated adaptive phase response. Of the five donors treated with KLH, two tested positive for IL-1β which is normally indicative of an innate phase response (**Figure 1A**). This could possibly be due to low levels of contaminating endotoxin or aggregates, which were not measured. Unstressed trastuzumab had minimal innate phase responses with only 1 of the 5 donors showing a positive response for IL-1β. Stir stressed trastuzumab aggregates had a positive response for IL-1β for all 5 donors (**Figure 1A**). Similar activation of IL-1β by aggregates has previously been reported (9).

Of the mAb1 containing process intermediate samples, the FNVI intermediate induced the highest response followed by the AEX and VF intermediates (**Figure 1A**). Four of the 5 donors treated with the FNVI intermediate were associated with a positive response for IL-1β (8-16-fold over baseline). This response decreased to 2/5 donors with an associated decrease in magnitude to almost 4-fold over baseline for the AEX product and 1/6 donors for the VF product with a very low magnitude of around 2-fold over baseline. A similar, low level (closer to 2-fold) IL-1β response was observed for 2/5 donors as well for the final formulated mAb1 drug product. The FNVI intermediate material also caused IL-6 and IL-10 responses for 2/5 donors.

Higher adaptive phase responses to antibodies observed in preclinical assessments have been implicated in driving high rates of anti-drug antibody formation in the clinic (10). For this study, the day 7 supernatants from donor PBMC challenged with the mAb1 drug products and upstream process intermediates were evaluated for cytokine profiles indicative of an adaptive T cell response. The PBMC were treated with PHA-P as a positive control for cytokine responses. All donors analyzed were screened for a positive response to KLH to confirm their robustness and ability to produce a naïve adaptive response *in vitro*. The unstressed and stir stressed trastuzumab were associated with cytokine responses for a few donors (2/5 donors and 1/5 donors respectively for IFN-γ and 0/5 donors and 3/5 donors for IL-2 (**Figure 1B**). This is in alignment with previous reports of cytokine responses to unstressed and stressed trastuzumab (10, 11).

The increase in innate responses caused by aggregated mAb1 containing samples did not translate to an increase in adaptive responses as no obvious difference was observed in the different process intermediate samples of mAb1. We did observe positive responses with a low magnitude on cytokine induction in up to 3/5 donors for mAb1 (**Figure 1B**). However, similar results were seen with the trastuzumab which has a low incidence of anti-drug antibodies in the clinic (prescribing information).

### Innate and Adaptive PBMC Responses to Spontaneously Occurring Aggregates in mAb1 Drug Product

Monitoring of mAb1 samples for stability at 5°C and 25°C led to the observation that this antibody forms aggregates spontaneously over time at these temperatures but displays little increase in aggregation when frozen at -80°C (**Tables 2**, **3**). These samples represent another example of spontaneously occurring antibody aggregates. We sought to determine if these spontaneously occurring aggregates containing samples elicited responses in the PBMC assay.

Innate phase responses were evaluated and are presented in **Figure 2A**. As expected, all 17 donors had a positive response to LPS (**Figure 2A**). 3/17 donors had positive IL-1β, IL-6, and TNF-α response to KLH. For trastuzumab, 3/17 donors had a positive IL-6 response. For the stir stressed trastuzumab, 12/17 had positive IL-1β and IL-6 responses while all 17 donors had a positive TNF-α response, thus indicating an innate phase response to the aggregates. The mAb1 drug product stored at 5°C had 3/17 donors with a positive response to IL-6. The mAb1 drug product stored at 25°C had 4/17 donors with positive responses to IL-1β, IL-6, and TNF-α. These cytokines were also produced in response to the upstream process material from the FNVI and AEX steps (**Figure 1A**). The drug product stored at -80°C resulted in 1/17 donors with positive responses to IL-1β. To summarize, an increase in innate phase responses to mAb1 samples trended with levels of aggregates.

For the adaptive phase responses, we analyzed human PBMC derived supernatants on day 7 from the same cells that were initially used for the 20 hr innate phase samples. The acceptance criterion for including the donors for analysis was their ability to respond to the positive controls PHA-P and KLH. IFN-γ was consistently increased in KLH treated samples and averaged around 5-fold above background levels (**Figure 2B**). The PBMC treated with the unstressed trastuzumab and stressed trastuzumab resulted in 4/17 donors and 5/17 donors with a positive IFN-γ response, respectively. The stirred stress trastuzumab resulted in higher number of donor responses associated with IL-13, IL-6, and TNF-α than the unstressed trastuzumab. It could be possible that monocytes are the source of IL-6 and TNF-α and the amounts detected at day 7 are carryover from the innate phase response. For mAb1 drug product stored at -80°C, a small number of donors elicited a positive IFN-γ and IL-13 response (3/17 for both analytes). The mAb1 drug products tested for stability at 12 months at 5°C and 25°C had no obvious differences in adaptive phase cytokines. For example, 4/17 donors with a positive response for IFN-γ for the 5°C 12-month drug product and 2/17 donors had a positive response for the same analyte for the 25°C 12-month drug product (**Figure 2B**).

### Sensitivity of the Reporter Cell Line THP-1 BLUE NFκB

*In vitro* assays can be used to assess immunogenicity risk due to changes in critical quality attributes of therapeutic proteins and different levels of therapeutic protein aggregates caused by changes in processing, formulation, and post fill conditions. Results from PBMC-based assays can be inconsistent due to the biological variability of human donors. Because of this variability multiple donors are required for representative results, which can be cost prohibitive as well as time consuming. Cell lines have several potential advantages for *in vitro* immunogenicity risk assessment: cost effective, typically more reproducible than primary cells, and more amenable to higher throughput. THP-1 BLUE NFκB cells are a commercially available monocytic cell line that is engineered to express the secreted embryonic alkaline phosphatase (SEAP) reporter gene under the control of NFκB enhancer elements. These cells are responsive to a variety of innate activating ligands including TLR2 and TLR4 agonists as well as Fcγ receptors (12). In our PBMC assay, innate phase chemokines are likely produced by monocytes and monocyte-derived antigen presenting cells. Therefore, we hypothesized that the THP-1 BLUE NFκB cell line could be used as a reliable method to assess immune activation due to antibody aggregates.

**Figure 3:**
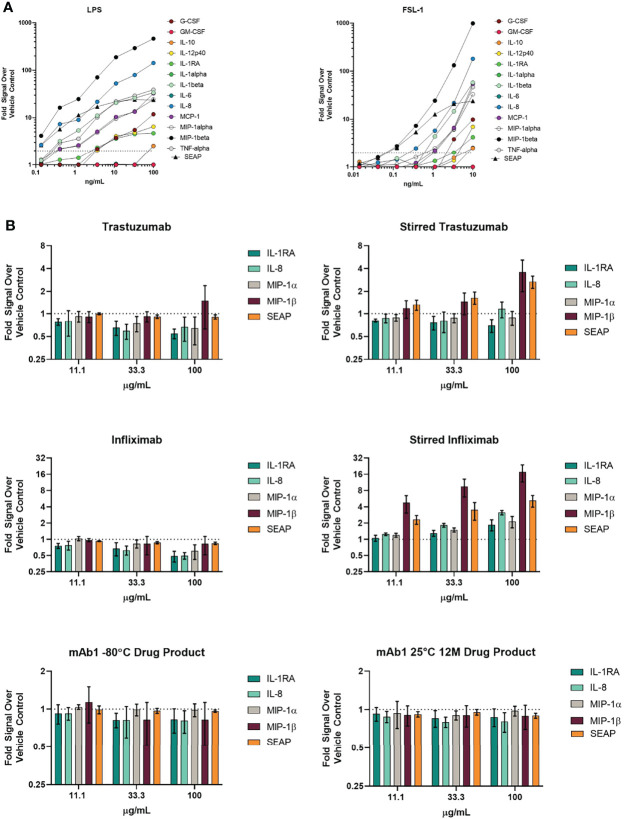
THP-1 BLUE NFκB responses to TLR ligands and various antibodies with different amounts of aggregation. **(A)** SEAP reporter gene production in response to TLR ligands are similar with regards to cytokine/chemokine production responses. THP-1 BLUE NFκB cells were treated with varying concentrations of the TLR4 ligand LPS or the TLR2/TLR6 ligand FSL-1. 24 hrs later, supernatant was removed for quantitation of SEAP activity and cytokines/chemokines. The results are normalized to the signal from the vehicle control. Results are the average of 3 independent experiments. **(B)** THP-1 BLUE NFκB responses to stressed antibodies. THP-1 BLUE NFκB cells were treated with stir stressed and unstressed trastuzumab, infliximab, or mAb1 drug product that had been stored at -80°C or at 25°C for 12 months. The cells were treated with three different concentrations. Results are the average of 3 independent experiments. Error bars represent S.E.M.

The responsiveness of this cell line to TLR2 and TLR4 agonists was assessed by comparing the SEAP reporter activity to the production of cytokines and chemokines. THP-1 BLUE NFκB SEAP responses to the TLR4 agonist LPS and the TLR2/6 agonist FSL-1 were similar to cytokine/chemokines responses from the same cell samples (**Figure 3A**). Therefore, the SEAP readout has potential to be sensitive enough to detect responses to aggregated antibodies.

We next evaluated if THP-1 BLUE NFκB responded to treatment with unstressed and stir stressed antibodies. Unstressed trastuzumab did not result in a 2-fold increase in cytokines, chemokines, and SEAP when compared to the vehicle treated control wells (**Figure 3B**). However, we did observe a greater than 2-fold increase in the levels of MIP-1β and SEAP The responses to unstressed and stressed infliximab were also evaluated. While the unstressed infliximab did not result in a greater than 2-fold increase SEAP, cytokines, or chemokines when compared to the vehicle treated wells, the stir stressed infliximab did result in higher levels of chemokines. Although THP-1 BLUE NFκB responded to the forced stressed mAb samples, the mAb1 12M 25°C sample with spontaneously forming aggregates did not yield a greater than 2-fold response for cytokines, chemokines or SEAP despite showing that some donors produced a response in the PBMC-based assay (**Figure 3B**).

## Discussion

Antibodies can form aggregates spontaneously during production, purification, drug product manufacturing or during storage. While robust formulation can stabilize therapeutic proteins, subsequent non-ideal storage and handling can lead to spontaneous formation of low levels of aggregates (13, 14). Additionally, forced stress conditions like pH and heat stress can lead to modifications like deamidation, oxidation, etc. that may also contribute to aggregation (15).

We and others have sought to evaluate the immunogenicity risk of therapeutic protein aggregates through *in vitro* and *in vivo* assays (10, 16, 17). Many of these experiments have used aggregates formed under high stress conditions as this is a convenient way to generate aggregates quickly and in large amounts. However, performing experiments with spontaneously occurring aggregates could be more informative and relevant. For instance, stressing antibodies or proteins in different ways can result in aggregates with differences in size, chemical modifications, particle surface hydrophobicity, and zeta potential that can result in differences in immunogenicity (15, 18). Protein aggregates vary in size and there have been conflicting reports as to which size of aggregates plays the biggest role in the induction of immune responses. Previously, fluorescent activated cell sorting (FACS) sorted particles that were enriched for 5 to 10 µm particles showed enhanced early and late-stage responses *in vitro* while fractions containing larger particles were less immunogenic (19). However, smaller sized particles may have better uptake by antigen presenting cells and increased drainage to lymph nodes (20, 21). Other reports suggest that submicron particles are better at uptake and eliciting an immune response in mice (16). *In vitro* studies have also suggested that submicron aggregates are better able to activate Fcγ receptors than aggregates in the micron range (22). While further studies on the size and properties will be beneficial to the understanding of antibody aggregate immunogenicity, it is more relevant to focus on the properties of spontaneously occurring aggregates.

Here we evaluated the *in vitro* immunogenicity of spontaneously occurring therapeutic antibody aggregates obtained from process intermediates and during drug product stability testing under storage at different temperature conditions. The distribution of the spontaneously formed aggregates skewed towards the 2-5 µm range as shown in **Tables 1**, **2**. The analytical characterization on the samples included assessment of particle size using MFI (**Tables 1**, **2**) and SEC-HPLC based high molecular weight species measurement (data not shown). The 12-month mAb1 25°C drug stability testing showed high molecular weight species being present at greater than 5% (data not shown). Micron sized particles, especially in the range of 2-10 µm, have demonstrated their ability to induce innate cytokine production and cellular uptake and subsequent presentation of therapeutic-derived protein by antigen presenting cells (9, 19, 23). Further mechanistic studies to determine if the innate cytokine production caused by micron sized particles is independent of cellular uptake are of interest.

We evaluated the immunogenicity potential of spontaneously forming aggregates using *in vitro* PBMC-based assays. *In vitro* immune activation signatures such as cytokine production and proliferation have been shown to align and trend well with immunogenicity/anti-drug antibody rates in the clinic (10, 24). For relative comparison with an antibody with a known clinical immunogenicity profile, we evaluated stressed forms of trastuzumab. The control form of trastuzumab likely formed aggregates after reconstitution, storage at -80°C and subsequent thawing as this product is lyophilized and is administered to the patient shortly after reconstitution (product prescribing information). Stir stressed trastuzumab had levels of aggregates several orders of magnitude higher than that of the spontaneously occurring aggregates.

We found varying responses of PBMC to the spontaneously occurring and the stir stressed aggregates. For the aggregates from mAb1 process intermediates, trastuzumab and stir stressed trastuzumab, the stir induced aggregated trastuzumab resulted in the highest percentage of donors responding as well as the highest average levels of cytokine/chemokine responses. Of the process related mAb1 intermediates, the FNVI sample had the highest level of aggregates and the highest innate phase PBMC responses. Antibody aggregates can occur during the low pH viral inactivation step and IgG4-based antibodies appear to be more susceptible to such pH-induced aggregation (3, 25–27). It is likely that mAb1, which is an IgG4, formed such aggregates. The mAb1 drug product stability samples also resulted in low levels of cytokine production indicative of innate phase responses and the magnitude and proportion of donors responding was much lower than with the stirred trastuzumab. The innate phase cytokine responses did not translate to cytokine response of an adaptive phase response in our assays. A low adaptive phase response to mAb1 may be due to it not containing MHC class II epitopes with high affinity and TCR-reactivity. Trastuzumab was found to have low rates of ADA formation in clinical trials (product insert for Herceptin and Herceptin Hylecta). While stir stressed trastuzumab resulted in increased innate immune response profiles for a majority of donors analysed in the *in vitro* assays, this did not always result in subsequent increases in adaptive phase responses. It has previously been shown that stir stressing an antibody can increase the percentage of donors whose PBMCs respond to different biologics (10). The adaptive phase responses observed for stir stressed trastuzumab be due to its intrinsic non-tolerant T-cell epitopes as well as the modification of epitopes by the post-translational modification of amino acids such as deamidation (28). Comparison of post-translational modifications of spontaneously forming and stress induced aggregates should be considered in future case studies. Also, the number of cells used in the assay may be not sufficient to consistently detect a response. This is supported by several publications that suggest the frequency of drug specific CD4+ T cells can be less than one per one million (29–31). However, determining the actual frequency of drug specific T cells is even more time consuming and labor intensive (31). While *in vitro* assays such as PBMC-based assays trend well with anti-drug antibody rates, such assays cannot predict the extent of maturity of immune response in clinic and are only representing the likelihood of a molecule to drive a T-cell dependent antibody response. Newer assays such as artificial lymph node models that can reconstruct the communication between antigen presenting cells and T cells over a long period may prove useful to understanding the humoral antibody immunogenicity (32, 33). While aggregates can potentially lead to increased ADA rates in patients, the innate phase response could play a role in infusion reactions and other adverse events (34–37).

Dendritic cells, have been shown to produce cytokines and upregulate cell surface markers that are associated with antigen presentation and co-stimulation of T-cells when exposed to antibody aggregates (38). Activation of cells has also trended with some monoclonal antibodies that have shown high immune responses *in vitro* and in the clinic (39). THP-1 cells have served as a cell line surrogate of antigen presenting cells. It has been demonstrated that THP-1 cells can take up therapeutic antibodies and produce inflammatory cytokines to several antibodies and that these cells were amenable to a high throughput format (40).

THP-1 BLUE NFκB cells may even provide for better automation as their readout for activation may be more convenient that measuring cytokines. While this cell line would not provide information on adaptive phase responses, it could provide insight on innate phase responses that could prime an adaptive phase response downstream. As to the mechanism for antibody aggregate induce immune activation, two reports suggest that TLR2 and TLR4 receptors play a role. Others have reported that an innate immune response modulating impurities can increase innate responses to antibodies (12, 41). Some innate immune response modulating impurities can signal through the TLR2 and TLR4 pathways as well. Cytokine production stimulated by antibody aggregates has been shown to be decreased in the presence of anti-TLR2 and anti-TLR4 antibodies (7, 9). Because THP-1 BLUE NFκB cells respond to these ligands and can mimic antigen presenting cells, we hypothesized that these cells would be able to respond to antibody aggregates. It should be noted that the parental THP-1 cells have been shown to express Fcγ receptors and may have a role in responses along with TLR4 (7, 12, 22). These stir stressed aggregates are generally analyzed in the micron size range.

It was observed that SEAP reporter gene responses to TLR2 and TLR4 ligands were similar to cytokine and chemokine responses assessed by Luminex in the supernatants. Additionally, the THP-1 BLUE NFκB cells were responsive to the stir stressed infliximab and trastuzumab but not to the unstressed forms. Interestingly, no increases in IL-1β, IL-6, IL-10, and TNF-α were observed despite previously reports of their increased production in response to aggregates (7, 12). However, both the previous study and our study did observe an increase in IL-8 for the aggregated antibody samples (7). We found THP-1 BLUE NFκB to be unresponsive to the 25°C 12-month mAb1 sample with low levels of spontaneously formed aggregates. We observed several donors with a response to the 25°C 12-month mAb1 with the PBMC assay. When comparing responses between the PBMC assay and the THP-1 BLUE NFκB, the PBMC assay was better able to respond to aggregates as evident from the reporter gene and cytokine/chemokine response profile readouts of THP-1 BLUE NFκB. Possible explanations for the lack of response of the THP-1 BLUE NFκB to the spontaneously occurring aggregates are differences in sensitivity to the number of aggregates or a difference in the properties of the aggregates themselves. Additionally, other cell populations in the PBMC assay could be contributing to the response. However, a study has shown that unmodified THP-1 cells showed different cytokine responses profiles to antibodies (40). The methods for this study had different culture conditions than what was used in our study and could be a potential explanation in the differences in results. This study also demonstrated that THP-1 can be used to monitor antibody internalization. This would be useful investigating aggregate size and uptake as well as the relationship between aggregate uptake and innate activation. Further evaluation of THP-1 BLUE NFκB to monitor responses to aggregates may be worth pursuing under different culture conditions.

This study focused on a case of a therapeutic antibody spontaneously forming aggregates during processing and stability testing and their *in vitro* immunogenicity. Such spontaneously forming aggregates occurring in lower levels than high stress induced samples can provide a more clinically relevant risk assessment. A number of donors were found to have innate phase cytokine responses when treated with spontaneously occurring aggregates. There are other indicators of innate phase activation such as upregulation of co-stimulatory molecules and phagocytic activity of antigen presenting cells that have been used in evaluating aggregate immunogenicity and should be included in future studies (11, 38, 40). There was a lack of an adaptive phase response to these spontaneously formed aggregates indicating a lack of priming of innate phase and propagation of this response to a T cell dependent immune response. The clinical relevance of such low levels of spontaneously formed aggregates observed during long term storage, pH changes or other handling related stress needs further evaluation. A data mining effort tracking the characterized clinical lots to each subject who receives the drug product can further help in understanding the risk posed by different levels of therapeutic protein aggregates and impact on safety and immunogenicity. Furthermore, physical characterization of the spontaneously occurring aggregates would be beneficial to determine if properties such as conformation, charge, reversibility or post-translational modifications correlate to immunogenic risk. Supplementary techniques such as size exclusion chromatography, dynamic light scattering, two-dimensional chromatography would be of benefit to future studies. In addition to this study, further examples of spontaneously forming antibody aggregates and their immunogenicity will allow for a better understanding of the risk they present to the development of safe and effective therapeutics.

## Data Availability Statement

The original contributions presented in the study are included in the article/supplementary material. Further inquiries can be directed to the corresponding author.

## Author Contributions

MS, SR, and SM performed experiments related to this paper. VJ, GS, SM, and MS contributed to the manuscript. All authors contributed to the article and approved the submitted version.

## Conflict of Interest

Authors MS, SR, SM and GS were employed by company Merck & Co., Inc. Author VJ was employed by company Bristol Myers Squibb.

## Publisher’s Note

All claims expressed in this article are solely those of the authors and do not necessarily represent those of their affiliated organizations, or those of the publisher, the editors and the reviewers. Any product that may be evaluated in this article, or claim that may be made by its manufacturer, is not guaranteed or endorsed by the publisher.
